# Met promotes the formation of double minute chromosomes induced by Sei-1 in NIH-3T3 murine fibroblasts

**DOI:** 10.18632/oncotarget.10994

**Published:** 2016-08-01

**Authors:** Yantao Bao, Jia Liu, Jia You, Di Wu, Yang Yu, Chang Liu, Lei Wang, Fei Wang, Lu Xu, Liqun Wang, Nan Wang, Xing Tian, Falin Wang, Hongbin Liang, Yating Gao, Xiaobo Cui, Guohua Ji, Jing Bai, Jingcui Yu, Xiangning Meng, Yan Jin, Wenjing Sun, Xin-yuan Guan, Chunyu Zhang, Songbin Fu

**Affiliations:** ^1^ Laboratory of Medical Genetics, Harbin Medical University, Harbin, China; ^2^ Department of Genetics and Eugenics, Maternity and Child Care Center of Qinghuangdao, Qinghuangdao, China; ^3^ Genetic Diagnosis Center, First People's Hospital of Yunnan Province, Yunnan, China; ^4^ Scientific Research Centre, Second Affiliated Hospital, Harbin Medical University, Harbin, China; ^5^ Department of Clinical Oncology, Faculty of Medicine, The University of Hong Kong, Hong Kong, China; ^6^ State Key Laboratory of Oncology in South China and Collaborative Innovation Center for Cancer Medicine, Sun Yat-sen University Cancer Center, Guangzhou, China; ^7^ Key Laboratory of Medical Genetics, Harbin Medical University, Heilongjiang Higher Education Institutions, Harbin, China

**Keywords:** Sei-1, in vivo passage, DMs, amplification, met

## Abstract

**Background:**

*Sei-1* is an oncogene capable of inducing double minute chromosomes (DMs) formation. DMs are hallmarks of amplification and contribute to oncogenesis. However, the mechanism of *Sei-1* inducing DMs formation remains unelucidated.

**Results:**

DMs formation significantly increased during serial passage *in vivo* and gradually decreased following culture *in vitro*. micro nuclei (MN) was found to be responsible for the reduction. Of the DMs-carrying genes, *Met* was found to be markedly amplified, overexpressed and highly correlated with DMs formation. Inhibition of Met signaling decreased the number of DMs and reduced the amplification of the DMs-carrying genes. We identified a 3.57Mb DMs representing the majority population, which consists of the 1.21 Mb AMP1 from locus 6qA2 and the 2.36 Mb AMP2 from locus 6qA2-3.

**Materials and Methods:**

We employed NIH-3T3 cell line with *Sei-1* overexpression to monitor and characterize DMs *in vivo* and *in vitro*. Array comparative genome hybridization (aCGH) and fluorescence *in situ* hybridization (FISH) were performed to reveal amplification regions and DMs-carrying genes. Metaphase spread was prepared to count the DMs. Western blot and Met inhibition rescue experiments were performed to examine for involvement of altered Met signaling in Sei-1 induced DMs. Genomic walking and PCR were adopted to reveal DMs structure.

**Conclusions:**

*Met* is an important promotor of DMs formation.

## INTRODUCTION

*Sei-1* is a cell cycle promotor and an identified oncogene [1] that is frequently amplified in different cancer types [1–4]. *Sei-1* is capable of promoting tumorigenesis by enhancing cell proliferation and prohibiting cell apoptosis [5, 6]. The involvement of *Sei-1* in chromosome instability has also been reported [3, 7]. Recent findings primarily focused on *Sei-1*'s oncogenic ability and potential tumor promoting pathways. Mechanisms that involve chromosome alterations that are inflicted by *Sei-1* were not fully elucidated.

Double minute chromosomes (DMs) are round-circle, acentric double-strand extra-chromosome DNA that usually exist in pairs. The formation of DMs is usually regarded as an important sign of genome instability [8–11]. DMs are more likely to harbor amplified genes [12]. The overexpressions of amplified genes carried on DMs are usually detected in different tumor types [13]. Genes carried on DMs, such as *MYC* and *DHFR* [14–16], are commonly identified as tumor-promoting genes that facilitate cancer progress and chemotherapy resistance [17–20]. DMs were also reported to be associated with tumor malignancy and poor prognoses [21–24]. As the primary manifestation of gene amplification, DMs carry varieties of oncogenes that may become potential and effective targets for clinical treatment.

Although DMs may be naturally lost during cell growth [25, 26], DMs can be maintained if DMs carried genes endowed advantages toward the host cells under certain selective pressures [25]. It may explain why oncogenes are often enriched on DMs in cancer. Previous studies demonstrate that a low dose of hydroxyurea (HU) treatment can reduce the number of DMs in various human carcinoma cell lines *in vitro* and *in vivo* [27–30] while ionizing radiation eliminates the amplified genes on DMs. Moreover, DMs could be captured inside MNs in tumor cells under HU and radiation treatment in numerous studies [31–33], and were exorcized from the cell with the MN capsules [22, 26]. Recent findings also revealed that the suppression of the DMs-carrying oncogenes can reduce the DMs population [34].

NIH-3T3 serves as a suitable model for the study of DMs in cancer biology. [35–38]. A previous study determined that *Sei-1* overexpressing NIH-3T3 cells not only gain a growth advantage but also produce DMs *in vivo* [7]. This finding aroused interest regarding how DMs are generated and confer malignant phenotypes. To obtain a better understanding, we re-established an NIH-3T3 model with *Sei-1* overexpression *in vitro* and *in vivo*. In this study, we innovatively investigated the mechanism of Sei-1 inducing the formation of DMs in nude mouse. We found the number of DMs induced by *Sei-1* was significantly increased during *in vivo* tissue passage but reduced during *in vitro* cell passage. Among the DMs-carrying genes, *Met* was the most prominently amplified and overexpressed. Met signaling pathway was shown to increase the *Sei-1*-induced DMs population, which indicated a novel function of the well-known oncogene *Met* to induce the formation of DMs. Additionally, a 3.57 Mb DMs structure was identified to represent the majority population of DMs with two amplicon fragments that were jointed with blunt ends.

## RESULTS

### Distinct evolution in DMs population between *in vivo* and *in vitro*

**Figure 1:**
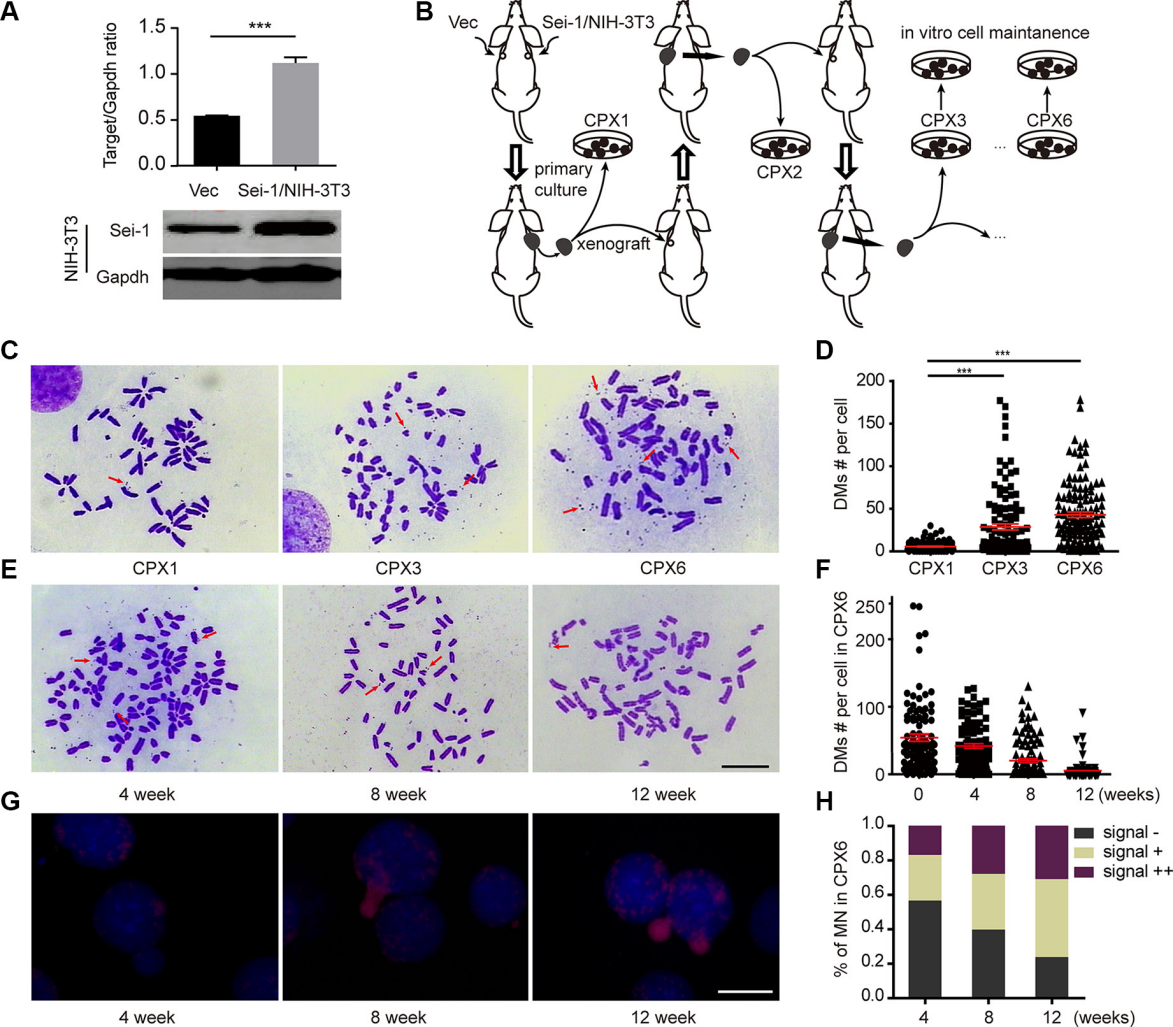
DMs population evolution *in vivo* and *in vitro* (**A**) Western blot results show *Sei-1* overexpression in NIH-3T3 cell clone Sei-1/NIH-3T3 compared with Vec. Upper, quantification of western blot results (*n* = 3), lower, representative western blot image. (**B**) Flow chart of *in vivo* and *in vitro* passage procedure. Three parallel procedures were employed, each mouse bore two tumor implants. (**C**) Metaphase spread images of primary cells CPX1, CPX3 and CPX6. Red arrows indicated the DMs. (**E**) Metaphase spread of CPX6 after four, eight, and 12 weeks of *in vitro* passage. (**D**) DMs count in CPX1, CPX3 and CPX6, for each group, *n* = 110 from three independent studies. (**F**) DMs number per karyotype in primary cells in four, eight, and 12 weeks of *in vitro* passage, *n* = 100 for each group. (**G**) FISH assays that show DMs signals in MNs after four, eight, and 12 weeks of *in vitro* passage. (**H**) The percentage of MNs with different staining states—“–”, no DMs signal, “+”, 0–50% DMs signal, and “++”–50%–100% DMs signals after four, eight, and 12 weeks of *in vitro* passage. Scale Bar, 10 μm. ***P < 0.001.

*Sei-1* and *null*-vector-transfected NIH-3T3 cell clone Sei-1/NIH-3T3 and Vec (Figure 1A) were subcutaneously injected into nude mice. Consistent with previous result [7], Sei-1/NIH-3T3 successfully formed a xenografted tumor, whereas Vec failed. The original generation achieved from the primary culture of cell clone Sei-1/NIH-3T3 formed a xenograft that was named CPX1; the second generation was named CPX2, the third generation was named CPX3…CPX6 (Figure 1B). DMs were scarcely detected in CPX1 but distinctly grew in CPX3 and CPX6 (Figure 1C and 1D). A distinct situation was observed after *in vitro* maintenance of CPX6. The number of DMs substantially decreased after eight weeks; in 12 weeks, the number of DMs was significantly reduced compared with the primary cells (Figure 1E and 1F). The DOP-PCR products of the microdissected DMs were probed and applied to CPX6 micronuclei spreads by FISH after different periods of *in vitro* maintenance. MNs that contain DMs signals were observed in these slides with the fluorescence signal substantially more condensed in MNs from early- to late-maintained cells (Figure 1G). In addition, the percentage of high DMs-signal harboring MN also increased from early- to late-maintained cells (Figure 1H). These results showed opposite changes in the DMs population between *in vivo* passage and *in vitro* passage. The results also indicated that MNs were responsible for the expulsion of DMs during *in vitro* cell culture.

### Genes carried on DMs were identified and overexpressed

**Figure 2:**
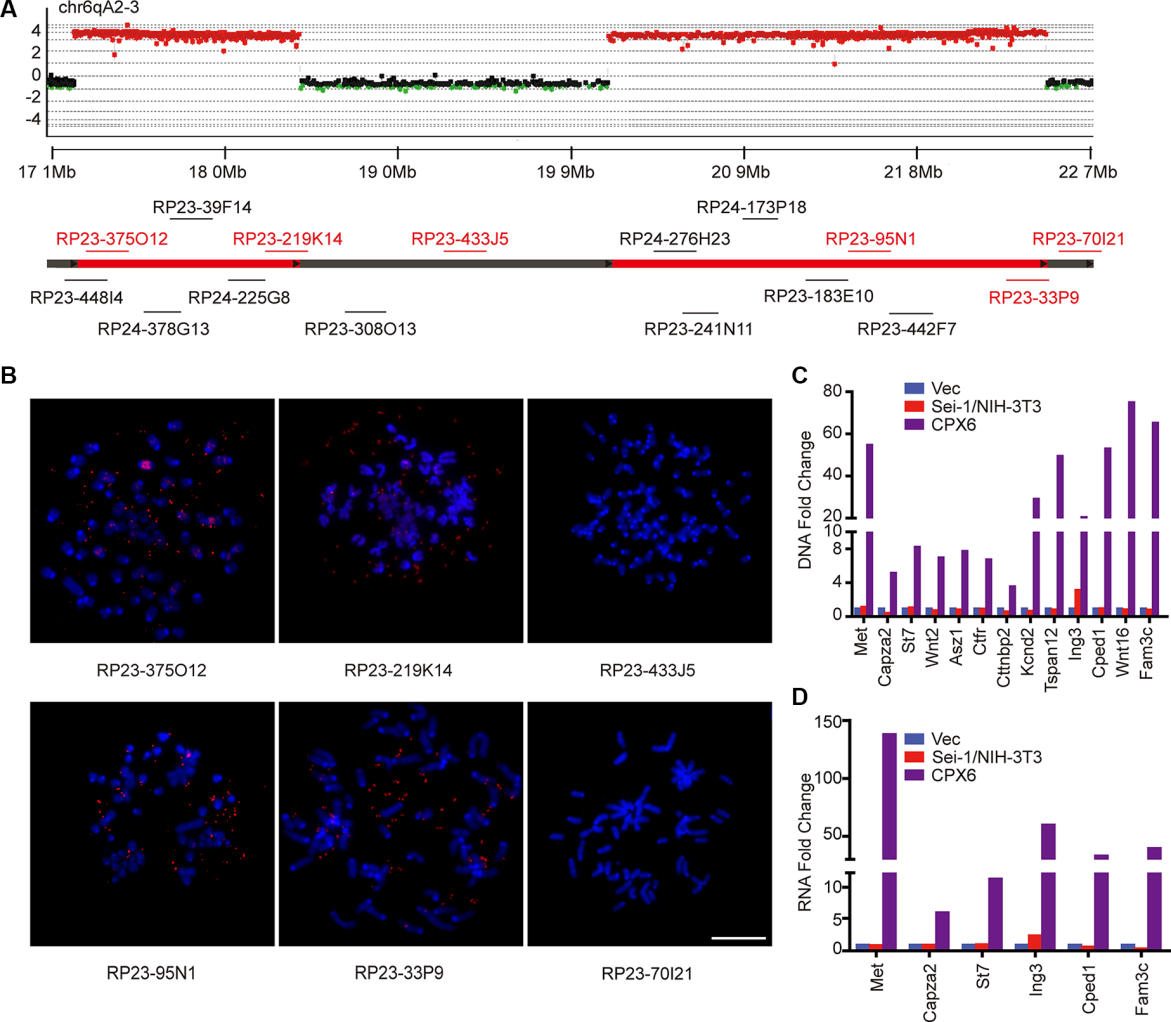
Identification of amplicons and amplified genes on DMs (**A**) Schematic of aCGH (Sei-1/NIH-3T3 *vs* CPX6) shows amplicons on chr6qA2-3; amplified BAC probes were represented as red dots, deleted probes were represented as green dots, and the normal probes as black dots. The BAC clones that were employed to validate the amplicons were labeled in corresponding positions of chromosomes. (**B**) Representative FISH images using the BAC clones that are shown in red; scale bar, 10 μm, quantitative real-time PCR results of DNA amplification level (**C**) and mRNA expression level (**D**) show amplification and overexpression of genes carried on DMs.

To elucidate the characteristics of DMs, a mouse aCGH was applied to find origins of DMs amplification. A genome overview of the aCGH results showed two distinct amplicons in the neighborhood on chromosome 6 (Supplementary Figure S1, Supplementary Table S1); no distinct amplified regions on other chromosomes were observed. A detailed aCGH probe-based graph revealed approximate sizes of the two amplicons, *e.g.*, 1.21 Mb for AMP1 and 2.36 Mb for AMP2, with identical amplification levels (Figure 2A). The FISH analysis with randomly chosen BAC probes that match the amplified region confirmed that the amplicons locate on the DMs (Figure 2A and 2B; Supplementary Table S2). The amplified genes were identified and subsequently confirmed to settle on DMs by FISH (Supplementary Table S3), *e.g., Met, Capza2, St7, Wnt2, Asz1, Ctfr, Cttnbp2* on AMP1 and *Kcnd2, Tspan12, Ing3, Cped1, Wnt16*, and *Fam3c* on AMP2. Quantitative real-time PCR confirmed that these genes were amplified on the DNA level and overexpressed in RNA (Figure 2C and 2D). *Met* was the most prominently overexpressed, which suggests that *Met* is the key activator of *Sei-1*-induced amplification. These results identified the exact genes carried on DMs; the majority of these genes were amplified and overexpressed, which indicates that these genes—especially *Met*—may actively enhance the tumorigenic and DMs-inducing potentials of *Sei-1*.

### Met signaling was activated as the DMs population increased

**Figure 3:**
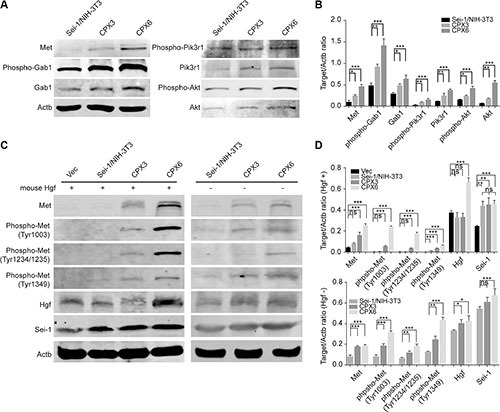
Met signaling was activated during *in vivo* passage (**A**) Western blot results show increases of Met/PI3K/Akt pathway from Sei-1/NIH-3T3, CPX3 to CPX6 during *in vivo* passage. (**B**) Quantification of protein level in A, *n* = 3. (**C**) Met phosphorylation was increased in the presence (“+”) of Hgf treatment from Sei-1/NIH-3T3, CPX3 to CPX6, at a dosage of 20 ng/ml, and absence (“–“) of Hgf treatment from Sei-1/NIH-3T3, CPX3 to CPX6 by *in vivo* passage. (**D**) Quantification of protein level in C with (+) or without (−) Hgf treatment, *n* = 3. *P < 0.05; **P < 0.01; ***P < 0.001.

The previous results elucidated an increased expression pattern of genes carried on DMs. *Met* was predominantly increased. *Met* was a well-depicted oncogene that drove carcinogenesis in many tumor types. Thus, *Met* was probably the most important gene amplified by *Sei-1*. The members involved in Met signaling pathway were detected using the western blot; a progressive increase of Met, Gab-1, Pik3r1, Akt protein and phosphorylation levels from Sei-1/NIH-3T3, CPX3 to CPX6 (Figure 3A and 3B) were discovered, which suggests that Met signaling pathway was activated during *in vivo* passage. Hepatocyte growth factor (Hgf) has been well elucidated to directly bind and motivate Met and its downward pathways. An alternative method of Met accumulation that causes self-phosphorylation and activation was also previously reported [39]. To determine what triggered Met signaling pathway, three activation sites of Met protein were tested using the western blot in *Sei-1* overexpressing the Sei-1/NIH-3T3 clone and primary cell pool CPX3, CPX6 with and without mouse recombinant Hgf treatment (Figure 3C and 3D). The results showed that Met was found to be activated even without Hgf treatment; however, the activation levels were lower than those of the corresponding Hgf treated groups. Besides, Sei-1 expression was found to be significantly higher in CPX6 compared to earlier generations of primary cells (Figure 3D and Supplementary Figure S2), suggesting that Sei-1 overexpression under *in vivo* condition remains consistent, although reasons for this elevated Sei-1 overexpression level was unclear. These findings suggested that the Met signaling pathway was activated as the number of DMs grew. It also suggested that Met primarily adopted self-activation manner during *in vivo* passage, although Hgf stimulation could still effectively activate Met.

### Inhibition of Met signaling pathway reduced the DMs population

**Figure 4:**
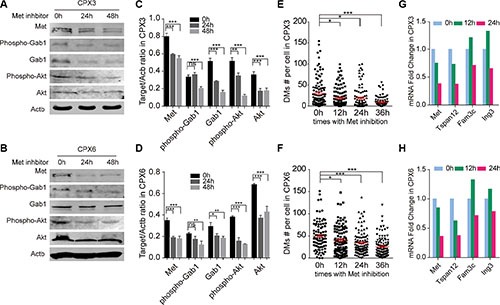
Met inhibition caused a loss in DMs Western blot results show effective inhibition in CPX3 (**A**) and CPX6 (**B**) for 24 and 48 hours with 4 × 10^−4^ μg/ml Met inhibitor treatment. Quantification data are shown in (**C**) (CPX3) and (**D**) (CPX6), *n* = 3. Scatter plot show a significant decrease in the DMs population 12, 24, and 36 hours with Met inhibition in (**E**) CPX3 and (**F**) CPX6, for each group, *n* = 100 from three independent studies. Quantitative real-time PCR results show decreased expression levels of DMs-carrying genes with Met inhibition in (**G**) CPX3 and (**H**) CPX6. A two-tailed student's *t-test* was employed as statistics; *P < 0.05, **P < 0.01, ***P < 0.001.

Met signaling activation was closely correlated to an increased number of DMs. A previous study revealed a decrease in the expression level of the DMs-carrying genes can decrease the number of DMs in cancer cells [34]. A recent study showed that DMs can be discharged from cells through MNs by inhibition of ERK activity, which causes a decrease in the DMs population [40]. We employed a Met-specific inhibitor—C_21_H_17_Cl_2_FN_2_O_2_—to separately block Met signaling in CPX3 and CPX6. The western blot verified the distinct inhibition of Met signaling in both CPX3 (Figure 4A and 4C) and CPX6 (Figure 4B and 4D) for 24 and 48 hours of drug treatment. Metaphase spreads were prepared 0, 12, 24, and 36 hours after Met inhibition for these two cell pools. DMs count demonstrated a significant reduction in the DMs population in both CPX3 and CPX6 cells at 24 hours and especially at 36 hours for inhibitor treatment (Figure 4E and 4F). Quantitative real-time PCR results revealed that the DMs-carrying genes (*Met, Tspan12, Fam3c*, and *Ing3*) were distinctly decreased 24 hours after Met inhibition as a result of the loss of DMs (Figure 4G and 4H). These findings conclusively showed that Met signaling inhibition reduces the DMs population and proved that Met signaling was responsible for the *Sei-1*-induced formation of DMs.

### AMP1 and AMP2 were directly jointed by blunt ends

**Figure 5:**
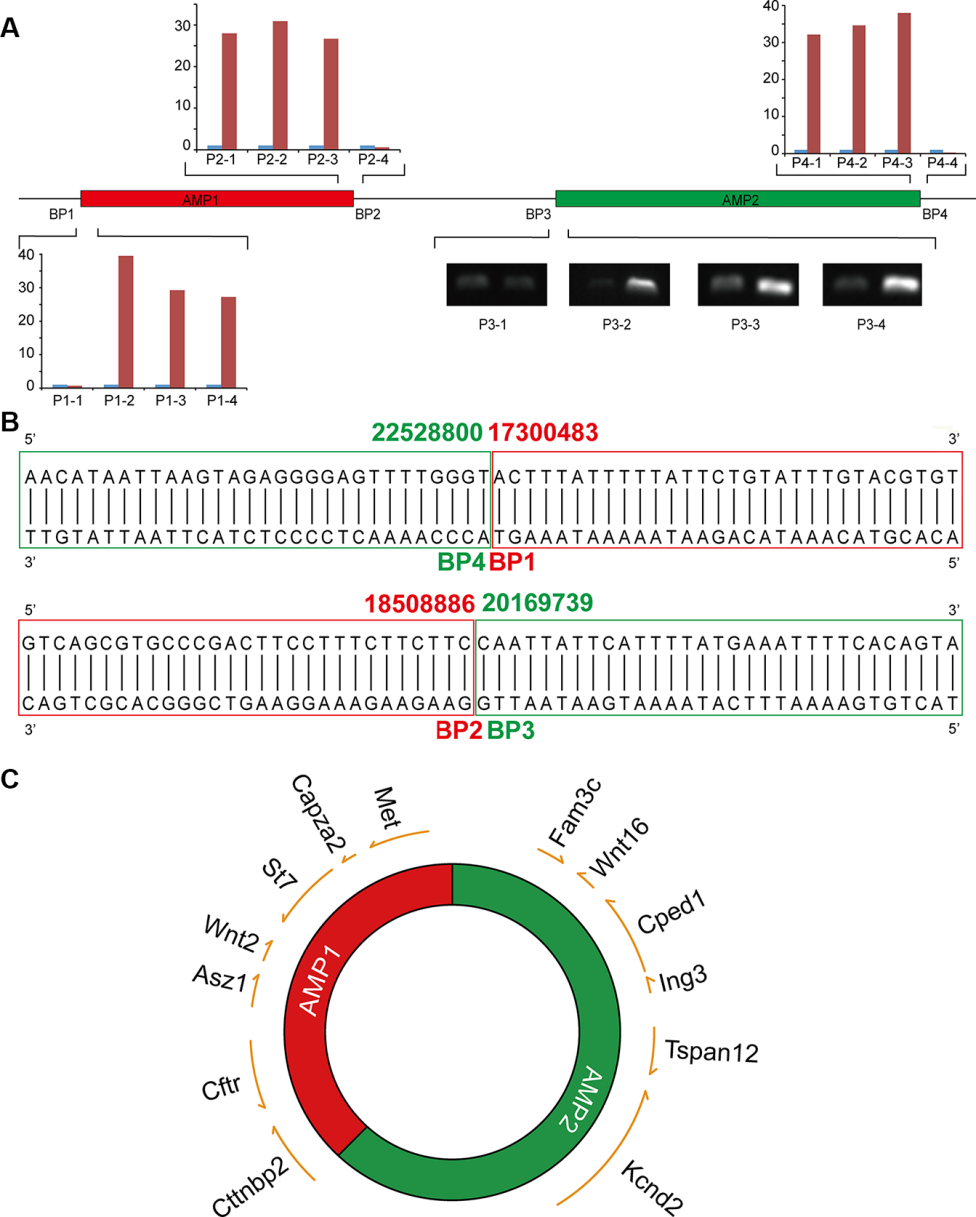
DMs structures consist of two individual amplicons (**A**) The boundaries of AMP1 and AMP2 were defined by quantitative real-time PCR for BP1, 2 and 4, and semi-quantitative PCR for BP3. (**B**) Blunt-end joints were identified via Sanger sequencing. AMP1 is shown in red and AMP2 is shown in green. (**C**) A schematic of DMs structures with DMs-carrying genes marked in the corresponding positions; the arrows indicate the transcription direction of each gene.

Well-delineated DMs structures may explain their evolutionary traces. Two juxtaposing amplicons (AMP1 and AMP2) with nearly identical copy changes were identified in this study: four breakpoints, namely, BP1, BP2 on the edge of AMP1, and BP3, BP4 on the edge of AMP2, which suggests a relatively simple DMs structure. Based on the aCGH results, a set of primers (Supplementary Table S4) were designed to narrow the gaps among amplicons (Figure 5A). Genomic walking technology was subsequently adopted to amplify the flanking fragments of BP1 and BP2, whose sequence information was subsequently achieved by Sanger sequencing. The sequencing results presented classic blunt-end junctions of both BP4-BP1 and BP2-BP3 (Figure 5B). Therefore, a diagram of a DMs structure with these two amplicons directly connected was constructed (Figure 5C). Dual-color FISH was conducted to confirm this structure with probes from two amplicons. We discovered that the majority of signals were perfectly merged; however, we did observe some cross-merged signals (Supplementary Figure S3), which suggests the minor existence of more complex structures that need to be studied and identified in a further study. In our model, we provide a relatively simple DMs structure induced by *Sei-1* that perfectly matches the aCGH results, which probably represents the majority population of DMs; however, other subtypes of DMs still existed due to heterogeneity of DMs.

## DISCUSSION

*Sei-1* is a newly discovered oncogene that is particularly found to be able to induce the formation of DMs. The biological connection between its oncogenic and DMs inducing abilities was barely explored. In this study, we innovatively observed the gain/loss of *Sei-1*-induced DMs to investigate oncogenic mechanism of *Sei-1*. Our study discovered *Met* as a dynamic responder to enhanced *Sei-1* oncogenic function. We uniquely found that *Met* was not only amplified in the form of DMs and overexpressed but also significantly involved in the formation of DMs itself, suggesting a novel function of *Met* to generate the DMs.

Clinical research reported DMs to worsen the cancer prognosis. [23, 24]. Evidence also proved tumor malignancy could be alleviated by eliminating DMs in ovarian cancer [34], nominating DMs elimination as promising strategy for therapy. Distinct evolution of DMs populations under different circumstances has been previously reported [41]. We observed vast increase of DMs *in vivo*, but progressively DMs lost during *in vitro* culture. A Darwinian selection pattern was previously implied to explain the increased number of DMs that harbor *MYCN* in neuroblastoma cells [42]. Recent report found the amplification-linked extrachromosomal mutations (ALEMs) that occurred on DMs were significantly lost after *in vitro* passages. They hypothesized that the number of DMs could change dynamically as a response to significant environmental changes [43]. *Sei-1-*induced DMs probably facilitate the survival of cells under *in vivo* pressure. *In vitro*, endogenous microenvironment pressure was retreated, and previous growth advantage conferred by DMs was lost. Therefore, DMs production was restricted, which caused a reduction in DMs populations. Interestingly, we observed the Sei- 1 overexpression level significantly higher in CPX6 compared to Sei-1/NIH-3T3, CPX1, CPX3 both in cells and tumor tissues (Figure 3D, Supplementary Figure S2), for which the reasons were unclear. We suspected that under *in vivo* condition Sei-1 expression level could somehow be enhanced to further increase the number of DMs.

DMs-carrying oncogenes were responsible for the malignant phenotype in cancer cells with DMs. These genes were most likely overexpressed but not always [44]. In this study, multiple oncogenes carried on DMs were both amplified and overexpressed, such as *Met*, *Wnt2*, *Tspan*12, *Wnt16* and *Fam3c*. *Met* can enhance various cancer malignancies [45–48] and concur poor diagnosis of cancer [49]. Here, we found *Met* overexpression to be the most prominent among all amplified genes, verifying *Me*t to be a key activator involved in *Sei-1*'s function in tumorigenesis and DMs induction. It's not hard to contemplate that *Sei-1* overexpression dynamically enhanced Met signaling pathway that could further obliged in a more aggressive tumor progress. *Met* has been found amplified on DMs in some tumors [15, 50] and cancer mouse models [51], which implies that *Met* is a hotspot of gene amplification and may participate in DMs formation. Nevertheless, participation of Met signaling pathway in accelerating DMs population was seldom reported before. Our results showed a positive correlation between self-activated Met signaling pathway and DMs formation. The inhibition of Met signaling pathway produced a significant loss of DMs, which proved that this pathway robustly promoted the formation of DMs. Therefore, Met signaling pathway may be a promising therapeutic target in treating tumors that contain DMs.

Episome excision model [52] and bridge-fusion-breakage (BFB) model [53] were two classical theories to explain DMs formation, though detailed mechanisms remain elusive. In this study, we located the boundaries of two amplicons which were jointed in blunt ends. Blunt-end joint suggested the involvement of a non-homology end junction (NHEJ) during the connection of the two amplicons. NHEJ was also responsible for other structure types, such as micro-homologies, anonymous insertions and palindrome sequences [54]. None of these structures was identified in this study.

Clinical samples were always found to bear compound genomic lesions. Complex genomic alteration could result from long-term accumulation as response to a series of selective pressure *in vivo* [44]. DMs may experience multiple structural alterations from the original structure to more complex structures [44]. The two identically amplified amplicons comprising the DMs highly matches the aCGH result. Dual-color FISH indicated that this simple structure of DMs was the most prevalent. The minority cross-unmerged signals indicated the existence of distinguished DMs structures, although these DMs all came from the two amplicons on chromosome 6, which suggests that the simple structure represents the primitive and ancestry DMs induced by Sei-1 while the otherwise structured DMs probably derive from the simple-structured DMs. The simple-structured DMs probably provide the universal template for subsequent DMs evolution. Due to the complexity of environment pressure and medical intervention experienced by the tumor cells clinically, direct investigation of DMs structures in clinical samples may be profoundly challenging. DMs originally induced by *Sei-1* was quite briefly structured, which suggested the *Sei-1* overexpression model could be adopted as promising material for studying the initial event during the formation of DMs.

## MATERIALS AND METHODS

### Ethic statement

Animal experiments in this manuscript have been conducted in accordance with the ethical standards and according to the Declaration of Helsinki and has been approved by the Ethnic Committee of Harbin Medical University (Approval identifier: HMUIRB20160009).

### Cell line and transfection

NIH-3T3, a mouse embryo originated fibroblastic cell line [55], was purchased from American Type Culture Collection (ATCC, Manassas, VA). The cell line was further authenticated through STR test by Microread (Beijing, China) and cultured with Dulbecco's modified Eagle's medium (DMEM; GIBCO, Carlsbad, CA) with 10% fetal bovine serum (PAA Laboratories GmbH, Pasching, Austria) in 37 °C incubator (Thermo Fisher, Waltham, MA) supplied with 5% CO2. Primary cell culture and cell passage was conducted using standard procedure. *Sei-1* CDS were cloned into pcDNA3.1(+) plasmid (Invitrogen, Auckland, New Zealand) and transfected into cells with Lipofectamine 2000 (Invitrogen).

### Animal experiment

Five-week-old Balb/c female nude mice were obtained from SLAC laboratory (Shanghai, China) and maintained within individual ventilated cages in SPF-degree conditions. The *s.c*. xenograft was conducted as previous [7], with each mouse bearing two points of cell injection (one for Vec and one for Sei-1/NIH-3T3). Three Sei-1/NIH-3T3 tumors formed. Three mice were used each time of tissue transplant as independent studies, each mouse bearing two identical implantations of Sei-1/NIH-3T3 tumor. When tumor size reached 2cm x 2cm, partial tumors from three mice were transplanted in three other mice separately while the rest from each mouse were primarily cultured to obtain cells for independent experiments. In tissue transplant, solid tumors were instantly separated after sacrifice and cut into ~1 mm³ dices. Five tumor dices were subcutaneously engrafted into another mouse.

### Metaphase spread preparation

Colcemid (Sigma-Aldrich, St. Louis, MO) was added to the medium of fast growing cells for 2 hours, which were trypsinized, washed with PBS, and subsequently centrifuged. Suspend pellets in pre-warmed 0.075 M KCl and incubate in 37 °C water bath for exact 14 min. Fixate the cells by fresh fixative solution (mixture of 3 methanol: 1 acetic acid) three times. Drop the cell suspension on a clean slide. The slides were dyed with Giemsa and photographed using Olympus BX41 microscope (Melville, NY, USA) equipped with JVC TK-C75U color video camera (JVC, Japan).

### Micronuclei spread preparation

A micronuclei spread was prepared without colcemid treatment. The protocol, including Giemsa dying and image capturing, was identical to metaphase spread.

### Fluorescence *in situ* hybridization (FISH)

BAC clones purchased from the BACPAC Resources Center (Children's Hospital Oakland, Oakland, CA) were labeled with fluorescence dUTPs and hybridized to metaphase/micronuclei spread as described [56]. The slides were counterstained with DAPI (Millipore, Billerica, MA) and observed and photographed using Leica DM-RXA2 fiuorescence microscope (Wetzlar, Germany). High-resolution photos were captured and analyzed using the MetaMorph Imaging System (Universal Imaging Corporation, West Chester, PA).

### Mouse genome aCGH

Genomic DNA samples were extracted from fresh xenograft tissue with the DNeasy Blood & Tissue Kit (Qiagen, Valencia, CA). Sample quality was guaranteed by Nanodrop (Thermo Fisher, Waltham, MA). The samples were properly predisposed and applied to a SurePrint G3 Mouse CGH Microarray (Agilent technologies, Santa Clara, CA). The procedures were conducted by Shanghai Biotechnology Co., Ltd. (Shanghai, China).

### DMs micro-dissection and degenerate oligonucleotide primed-polymerase chain reaction (DOP-PCR)

The DMs were micro-dissected from NIH-3T3 metaphase karyotype that were prepared as previously described [56]. The samples were administered with Topoisomerase I (Promega Corp., Madison, WI) and amplified using Sequenase version 2.0 DNA polymerase (United States Biochemical Corporation, Cleveland, OH) and a commonly adopted degenerate primer (5′-CCGACTCGAGNNNNNNATGTGG-3′).

### Semi-quantitative PCR and real-time quantitative PCR

Genomic DNA and RNA samples were extracted by QIAamp^®^ DNA Mini and Blood Mini Kit (Qiagen) and Trizol (Roche, Alameda, CA). cDNA samples were obtained using Transcriptor First Strand cDNA Synthesis Kit (Roche). For quantitative real-time PCR, the samples were tested using the LightCycler^®^ 480 instrument (Roche) and corresponding SYBR Green I master (Roche). Semi-quantitative PCR was performed as described [57].

### Genome walking

The Universal Genomic Walker Kit (Clontech Laboratories., Mountain View, CA) was employed according to the manufacture instructions. The PCR products were sequenced and aligned with the mouse genome using the BLAT tool online from UCSC. The gene-specific primers (GSP) for BP1 were GSP1, 5′-TTGGTATTTATTCACGACTTTGATAC-3′, GSP2, 5′-GTCATGTACTCTGTTAAGACTGCTTT-3′. The GSP primers for BP2 were GSP1, 5′-AACAGATGAA AAAAAGTTGAGAGGCT-3′, GSP2, 5′- AACAGATGA AAAAAAGTTGAGAGGCT-3′

### Western blot

Cells were lysed by RIPA buffer and sonication, and centrifuged at 12000 g 4°C for 30 minutes. Supernatant protein was separated by SDS-polyacrylamide gel, and transferred to PVDF membrane, which was scanned by Odyssey Imaging System (Li-COR, Lincoln, NE) after incubation of corresponding antibodies. Image J Software was used for quantification.

### Antibodies and reagents

Antibodies against Gab1—phospho-Gab1, Met—Phospho-Met, pan-AKT—Phospho-Akt, PI3K, and Phospho-PI3K were purchased from Cell Signaling (Danvers, MA). Antibodies against Hgf were purchased from Thermo Fisher (Waltham, MA). Anti-mouse and anti-rabbit antibodies were obtained from Rockland Immunochemicals (Gilbertsville, PA, USA). Mouse recombinant Hgf and c-Met Kinase Inhibitor III were purchased from Millipore (Billerica, MA).

### Statistical analysis

Data of the DMs counts and Western blot coming evenly from three parallel studies were presented as the mean ± S.D. All experiments were independently repeated three times. Two-tailed Student's *t-test* as well as one-way ANOVA with SNK test were employed as the statistics.

## SUPPLEMENTARY MATERIALS FIGURES AND TABLES



## References

[1] Tang TC, Sham JS, Xie D, Fang Y, Huo KK, Wu QL, Guan XY (2002). Identification of a candidate oncogene SEI-1 within a minimal amplified region at 19q13. 1 in ovarian cancer cell lines. Cancer Res.

[2] Darwish H, Cho JM, Loignon M, Alaoui-Jamali MA (2007). Overexpression of SERTAD3, a putative oncogene located within the 19q13 amplicon, induces E2F activity and promotes tumor growth. Oncogene.

[3] Li Y, Nie CJ, Hu L, Qin Y, Liu HB, Zeng TT, Chen L, Fu L, Deng W, Chen SP, Jia WH, Zhang C, Xie D (2010). Characterization of a novel mechanism of genomic instability involving the SEI1/SET/NM23H1 pathway in esophageal cancers. Cancer Res.

[4] Gronwald J, Jauch A, Cybulski C, Schoell B, Bohm-Steuer B, Lener M, Grabowska E, Gorski B, Jakubowska A, Domagala W, Chosia M, Scott RJ, Lubinski J (2005). Comparison of genomic abnormalities between BRCAX and sporadic breast cancers studied by comparative genomic hybridization. Int J Cancer.

[5] cHong SW, Kim CJ, Park WS, Shin JS, Lee SD, Ko SG, Jung SI, Park IC, An SK, Lee WK, Lee WJ, Jin DH, Lee MS (2009). p34SEI-1 inhibits apoptosis through the stabilization of the X-linked inhibitor of apoptosis protein: p34SEI-1 as a novel target for anti-breast cancer strategies. Cancer Res.

[6] Hong SW, Shin JS, Lee YM, Kim DG, Lee SY, Yoon DH, Jung SY, Hwang JJ, Lee SJ, Cho DH, Hong YS, Kim TW, Jin DH (2011). p34 (SEI-1) inhibits ROS-induced cell death through suppression of ASK1. Cancer Biol Ther.

[7] Tang DJ, Hu L, Xie D, Wu QL, Fang Y, Zeng Y, Sham JS, Guan XY (2005). Oncogenic transformation by SEI-1 is associated with chromosomal instability. Cancer Res.

[8] Zimonjic DB, Zhang H, Shan Z, Factor V, Trent J, Thorgeirsson SS, Popescu NC (2001). DNA amplification associated with double minutes originating from chromosome 19 in mouse hepatocellular carcinoma. Cytogenet Cell Genet.

[9] Montagna C, Andrechek ER, Padilla-Nash H, Muller WJ, Ried T (2002). Centrosome abnormalities, recurring deletions of chromosome 4, and genomic amplification of HER2/neu define mouse mammary gland adenocarcinomas induced by mutant HER2/neu. Oncogene.

[10] Papachristou F, Simopoulou M, Touloupidis S, Tsalikidis C, Sofikitis N, Lialiaris T (2008). DNA damage and chromosomal aberrations in various types of male factor infertility. Fertil Steril.

[11] Boone CW, Kelloff GJ (1994). Development of surrogate endpoint biomarkers for clinical trials of cancer chemopreventive agents: relationships to fundamental properties of preinvasive (intraepithelial) neoplasia. J Cell Biochem Suppl.

[12] Benner SE, Wahl GM, Von Hoff DD (1991). Double minute chromosomes and homogeneously staining regions in tumors taken directly from patients versus in human tumor cell lines. Anti-Cancer Drug.

[13] Von Hoff DD, Needham-VanDevanter DR, Yucel J, Windle BE, Wahl GM (1988). Amplified human MYC oncogenes localized to replicating submicroscopic circular DNA molecules. P Natl Acad Sci USA.

[14] Seruca R, Suijkerbuijk RF, Gartner F, Criado B, Veiga I, Olde-Weghuis D, David L, Castedo S, Sobrinho-Simoes M (1995). Increasing levels of MYC and MET co-amplification during tumor progression of a case of gastric cancer. Cancer Genet Cytogen.

[15] Hara T, Ooi A, Kobayashi M, Mai M, Yanagihara K, Nakanishi I (1998). Amplification of c-myc, K-sam, and c-met in gastric cancers: detection by fluorescence *in situ* hybridization. Lab Invest.

[16] Morales C, Ribas M, Aiza G, Peinado MA (2005). Genetic determinants of methotrexate responsiveness and resistance in colon cancer cells. Oncogene.

[17] Gebhart E (2005). Double minutes, cytogenetic equivalents of gene amplification, in human neoplasia - a review. Clin Transl Oncol.

[18] Alitalo K (1984). Amplification of cellular oncogenes in cancer cells. Med Biol.

[19] Kaye S, Merry S (1985). Tumour cell resistance to anthracyclines—a review. Cancer Chemoth Pharm.

[20] Lin CT, Lyu YL, Xiao H, Lin WH, Whang-Peng J (2001). Suppression of gene amplification and chromosomal DNA integration by the DNA mismatch repair system. Nucleic Acids Res.

[21] Gebhart E, Bruderlein S, Tulusan AH, von Maillot K, Birkmann J (1984). Incidence of double minutes, cytogenetic equivalents of gene amplification, in human carcinoma cells. Int J Cancer.

[22] Cuthbert G, Thompson K, McCullough S, Watmore A, Dickinson H, Telford N, Mugneret F, Harrison C, Griffiths M, Bown N (2000). MLL amplification in acute leukaemia: a United Kingdom Cancer Cytogenetics Group (UKCCG) study. Leukemia.

[23] Thomas L, Stamberg J, Gojo I, Ning Y, Rapoport AP (2004). Double minute chromosomes in monoblastic (M5) and myeloblastic (M2) acute myeloid leukemia: two case reports and a review of literature. Am J Hematol.

[24] Li YS (1983). Double minutes in acute myeloid leukemia. Int J Cancer.

[25] Kuttler F, Mai S (2007). Formation of non-random extrachromosomal elements during development, differentiation and oncogenesis. Semin Cancer Biol.

[26] Shimizu N (2009). Extrachromosomal double minutes and chromosomal homogeneously staining regions as probes for chromosome research. Cytogenet Genome Res.

[27] Raymond E, Faivre S, Weiss G, McGill J, Davidson K, Izbicka E, Kuhn JG, Allred C, Clark GM, Von Hoff DD (2001). Effects of hydroxyurea on extrachromosomal DNA in patients with advanced ovarian carcinomas. Clinical Cancer Res.

[28] Nevaldine BH, Rizwana R, Hahn PJ (1999). Differential sensitivity of double minute chromosomes to hydroxyurea treatment in cultured methotrexate-resistant mouse cells. Mutat Res.

[29] Canute GW, Longo SL, Longo JA, Shetler MM, Coyle TE, Winfield JA, Hahn PJ (1998). The hydroxyurea-induced loss of double-minute chromosomes containing amplified epidermal growth factor receptor genes reduces the tumorigenicity and growth of human glioblastoma multiforme. Neurosurgery.

[30] Von Hoff DD, McGill JR, Forseth BJ, Davidson KK, Bradley TP, Van Devanter DR, Wahl GM (1992). Elimination of extrachromosomally amplified MYC genes from human tumor cells reduces their tumorigenicity. P Natl Acad Sci USA.

[31] Shimizu N, Kanda T, Wahl GM (1996). Selective capture of acentric fragments by micronuclei provides a rapid method for purifying extrachromosomally amplified DNA. Nat Genet.

[32] Valent A, Benard J, Clausse B, Barrois M, Valteau-Couanet D, Terrier-Lacombe MJ, Spengler B, Bernheim A (2001). *In vivo* elimination of acentric double minutes containing amplified MYCN from neuroblastoma tumor cells through the formation of micronuclei. Am J Pathol.

[33] Sanchez AM (1998). Fractionated Ionizing Radiation Accelerates Loss of Amplified MDR1 Genes Harbored by Extrachromosomal DNA in Tumor Cells. Cancer Res.

[34] Ji W, Bian Z, Yu Y, Yuan C, Liu Y, Yu L, Li C, Zhu J, Jia X, Guan R, Zhang C, Meng X, Jin Y (2014). Expulsion of micronuclei containing amplified genes contributes to a decrease in double minute chromosomes from malignant tumor cells. Int J Cancer.

[35] Murray MJ, Kaufman RJ, Latt SA, Weinberg RA (1983). Construction and use of a dominant, selectable marker: a Harvey sarcoma virus-dihydrofolate reductase chimera. Mol Cell Biol.

[36] Seo J, Chung YS, Sharma GG, Moon E, Burack WR, Pandita TK, Choi K (2005). Cdt1 transgenic mice develop lymphoblastic lymphoma in the absence of p53. Oncogene.

[37] Cahilly-Snyder L, Yang-Feng T, Francke U, George DL (1987). Molecular analysis and chromosomal mapping of amplified genes isolated from a transformed mouse 3T3 cell line. Somat Cell Mol Genet.

[38] Zhang CY, Feng YX, Yu Y, Sun WJ, Bai J, Chen F, Fu SB (2006). The molecular mechanism of resistance to methotrexate in mouse methotrexate-resistant cells by cancer drug resistance and metabolism SuperArray. Basic Clin Pharmacol.

[39] Varkaris A, Gaur S, Parikh NU, Song JH, Dayyani F, Jin JK, Logothetis CJ, Gallick GE (2013). Ligand-independent activation of MET through IGF-1/IGF-1R signaling. Int J Cancer.

[40] Sun W, Quan C, Huang Y, Ji W, Yu L, Li X, Zhang Y, Zheng Z, Zou H, Li Q, Xu P, Feng Y, Li L (2014). Constitutive ERK1/2 activation contributes to production of double minute chromosomes in tumour cells. J Pathol.

[41] Haber DA, Schimke RT (1981). Unstable amplification of an altered dihydrofolate reductase gene associated with double-minute chromosomes. Cell.

[42] Lundberg G, Rosengren AH, Hakanson U, Stewenius H, Jin Y, Stewenius Y, Pahlman S, Gisselsson D (2008). Binomial mitotic segregation of MYCN-carrying double minutes in neuroblastoma illustrates the role of randomness in oncogene amplification. PloS one.

[43] Nikolaev S, Santoni F, Garieri M, Makrythanasis P, Falconnet E, Guipponi M, Vannier A, Radovanovic I, Bena F, Forestier F, Schaller K, Dutoit V, Clement-Schatlo V (2014). Extrachromosomal driver mutations in glioblastoma and low-grade glioma. Nat Commun.

[44] L'Abbate A, Macchia G, D'Addabbo P, Lonoce A, Tolomeo D, Trombetta D, Kok K, Bartenhagen C, Whelan CW, Palumbo O, Severgnini M, Cifola I, Dugas M (2014). Genomic organization and evolution of double minutes/homogeneously staining regions with MYC amplification in human cancer. Nucleic Acids Res.

[45] Weidner K, M S, W B (1993). The Met Receptor Tyrosine Kinase Transduces Motility, Proliferation, And Morphogenic Signals Of Scatter Factor/Hepatocyte Growth Factor In Epithelial Cells. J Cell Biol.

[46] Tsarfaty I, Rong S (1994). The Met Proto-Oncogene Mesenchymal to Epithelial Cell Conversion. Science.

[47] Sun S, Wang Z (2011). Head neck squamous cell carcinoma c-Met(+) cells display cancer stem cell properties and are responsible for cisplatin-resistance and metastasis. Int J Cancer.

[48] Boccaccio C, Luraghi P, Comoglio PM, Boccaccio C (2014). MET-mediated resistance to EGFR inhibitors: an old liaison rooted in colorectal cancer stem cells. Cancer Res.

[49] Kim YJ, Choi JS, Seo J, Song JY, Eun Lee S, Kwon MJ, Kwon MJ, Kundu J, Jung K, Oh E (2014). MET is a potential target for use in combination therapy with EGFR inhibition in triple-negative/basal-like breast cancer. Int J Cancer.

[50] Collins VP (1995). Gene amplification in human gliomas. Glia.

[51] Smolen GA, Muir B, Mohapatra G, Barmettler A, Kim WJ, Rivera MN, Haserlat SM, Okimoto RA, Kwak E, Dahiya S, Garber JE, Bell DW, Sgroi DC (2006). Frequent met oncogene amplification in a Brca1/Trp53 mouse model of mammary tumorigenesis. Cancer Res.

[52] Carroll SM, DeRose ML, Gaudray P, Moore CM, Needham-Vandevanter DR, Von Hoff DD, Wahl GM (1988). Double minute chromosomes can be produced from precursors derived from a chromosomal deletion. Mol Cell Biol.

[53] Murnane JP, Sabatier L (2004). Chromosome rearrangements resulting from telomere dysfunction and their role in cancer. BioEssays.

[54] Narayanan V, Mieczkowski PA, Kim HM, Petes TD, Lobachev KS (2006). The pattern of gene amplification is determined by the chromosomal location of hairpin-capped breaks. Cell.

[55] Jainchill JL, Aaronson SA, Todaro GJ (1969). Murine sarcoma and leukemia viruses: assay using clonal lines of contact-inhibited mouse cells. J Virol.

[56] Guan XY, Trent JM, Meltzer PS (1993). Generation of band-specific painting probes from a single microdissected chromosome. Hum Mol Genet.

[57] Zhu J, Yu Y, Meng X, Fan Y, Zhang Y, Zhou C, Yue Z, Jin Y, Zhang C, Yu L, Ji W, Jia X, Guan R (2013). *De novo*-generated small palindromes are characteristic of amplicon boundary junction of double minutes. Int J Cancer.

