# Supplementary material for: Met promotes the formation of double minute chromosomes induced by Sei-1 in NIH-3T3 murine fibroblasts
Source: Oncotarget. 2016 Aug 1;7(35):56664–75. doi: 10.18632/oncotarget.10994 (PMC5302943; doi:10.18632/oncotarget.10994)
Supplement: Supplementary file 1 [file oncotarget-07-56664-s001.pdf]

Met promotes the formation of double minute chromosomes induced by Sei-1 in NIH-3T3 murine fibroblasts

Supplementary Materials

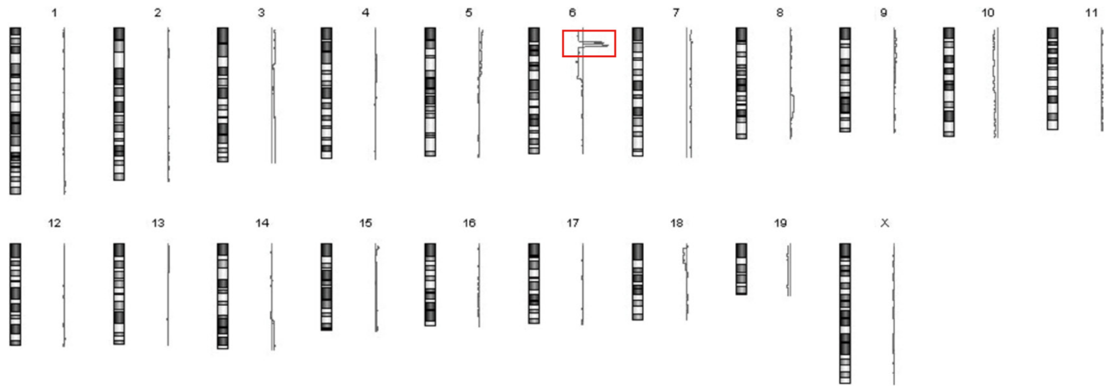

Supplementary Figure S1: Genome-wide overview of aCGH results. Red rectangle indicates two neighboring amplicons on chr 6. No distinct genomic alteration was observed elsewhere.

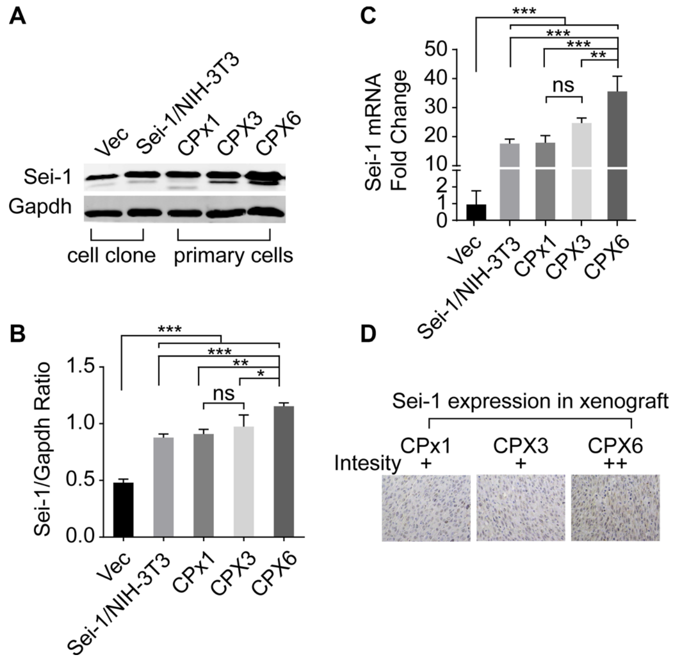

Supplementary Figure S2: Enhanced Sei-1 expression during *in vivo* passage. (**A**) Western Blot results that show Sei-1 protein expression in cell clones Vec, Sei-1/NIH-3T3 and primary cells CPX1, CPX3, CPX6. (**B**) Quantification of western blot results in (A). (**C**) Real-time PCR results show Sei-1 expression level in cell clones Vec, Sei-1/NIH-3T3 and primary cells CPX1, CPX3, CPX6. (**D**) Immunohistochemistry results that show Sei-1 staining in tumor tissues of CPX1, CPX3, CPX6, the percentages of positively stained cells were all more than 85% in three tumors, staining intensity was categorized as + (lower) and ++ (higher), $*P < 0.05$, $**P < 0.01$, $***P < 0.001$.

Supplementary Table S1: Interval-based aCGH results of the amplification of two amplicons

AberrationNo	Chr	Cytoband	Start	Stop	#Probes	Amplification
151	chr6	qA1–qA2	3272657	16807910	4404	0
152	chr6	qA2	16809298	18990859	953	264.685394
153	chr6	qA2	18994707	19694581	106	0
154	chr6	qA2–qA3.1	19703287	22983851	1063	283.55191
155	chr6	qA3.1–qB3	22985558	62114316	16677	0
156	chr6	qC1	63030049	63058537	5	0
157	chr6	qC1	63194750	63312030	71	0
158	chr6	qC1	63553927	63557644	3	0
159	chr6	qC2	75692682	75765769	14	0
160	chr6	qE1	103960104	104722531	235	0
161	chr6	qE1	108078162	108123542	31	4.084739
162	chr6	qE2	108620752	108738096	36	4.18692
163	chr6	qG2	141139070	141195567	19	0
164	chr6	qG2	141302967	141341464	23	0
165	chr6	qG3	147117277	147201712	29	0

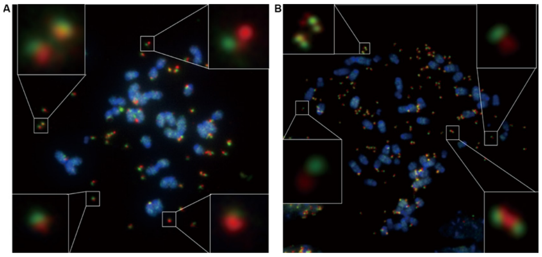

Supplementary Figure S3: Crossed and unmerged dual-color FISH signals in two representative images. The following probes were employed: (**A**) Green, RP23-95N1 (AMP2); Red, RP23-375O12 (AMP1) and (**B**) Green, RP23-219K14 (AMP1), Red, RP24-173P18 (AMP2),

Supplementary Table S2: BAC clones used to confirm DMs amplification

	BAC CLONE		Position		Amplified as DMs	genes involved
			start	end		
prior	RP23-448I4	chr6	17,137,101	17,316,534	–	CAV1 CAV2
AMP1	RP23-375O12	chr6	17,312,272	17,460,230	+	MET
	RP24-378G13	chr6	17,545,422	17,683,046	+	CAPAZ ST7
	RP23-39F14	chr6	17,875,942	18,085,740	+	St7,Wnt2, Asz1
	RP24-225G8	chr6	18,077,831	18,244,393	+	CTFR
	RP23-219K14	chr6	18,293,564	18,517,211	+	CTTNBP2
Intervel	RP23-308O13	chr6	18,691,468	18,900,437	–	—
	RP23-433J5	chr6	19,254,947	19,434,519	–	—
AMP2	RP24-276H23	chr6	20,451,044	20,617,168	+	—
	RP23-241N11	chr6	20,546,223	20,723,095	+	—
	RP24-173P18	chr6	20,893,261	21,049,969	+	—
	RP23-183E10	chr6	21,241,551	21,452,546	+	KCND2
	RP23-95N1	chr6	21,462,945	21,702,553	+	KCND2
	RP23-442F7	chr6	21,681,522	21,869,251	+	TSPAN12
	RP23-33P9	chr6	22,432,782	22,652,781	+	FAM3C
posterior	RP23-70I21	chr6	22,629,815	22,799,558	–	—

Supplementary Table S3: Amplified genes carried on DMs

	Official Symbol	Genebank accession	human	mouse	Cancer related
AMP1	Met	NM_008591.2	7q31	6 A2	Oncogene
	Capza2	NM_007604.2	7q31	6 A2	—
	St7	NM_001083315.2	7q31	6 A2	Tumor suppressor
	Wnt2	NM_023653.5	7q31	6 A2	proto-oncogene
	Asz1	NM_023729.3	7q31	6 A2	linked
	Cftr	NM_021050.2	7q31	6 A2	—
	cttnbp2	NM_080285.1	7q31	6 A2	—
AMP2	Kcnd2	NM_019697.3	7q31	6 A2–A3.1	linked
	Tspan12	NM_173007.3	7q31	6 A3	tumor promotor
	Ing3	NM_023626.4	7q31	6 A3	Tumor suppressor
	Cped1	NM_001081351.1	7q31	6 A3	—
	Wnt16	NM_053116.4	7q31	6 A3	tumor promotor
	Fam3c	NM_138587.4	7q31	6 A3	tumor promotor

Supplementary Table S4: Primers for quantitative real-time PCR and semi-quantitative PCR in boundary determination

Position	Name		Primer sequences	Length	Ranges
BP1	P1-1	Forward	TCAGAGCCCTACAACAGC	18	17299856–17299873
		Reverse	TCCACTATGCCCTTTGAG	18	17299991–17300008
	P1-2	Forward	TCACATGTCACTGCTATTG	19	17303207–17303225
		Reverse	GCAATCATCATCCTATAATC	20	17303338–17303357
	P1-3	Forward	ACTGTGTTCACTGCTGCTC	19	17304252–17304270
		Reverse	TTCCCATGTGTTCCTAGC	18	17304392–17304409
	P1-4	Forward	GCTGTGGTCCTAAATCCTGTG	21	17306125–17306145
		Reverse	GTATAAGGTGCGGGTGAAGG	20	17306256–17306275
BP2	P2-1	Forward	GGCAACATCTACACCTAAG	19	18461851–18461869
		Reverse	GTAGGCAGTATGGAAAGC	18	18461998–18462015
	P2-2	Forward	CCAACAATCATAACAACAAC	20	18477757–18477776
		Reverse	AAGCACTCATTGTTAGCAG	19	18477941–18477959
	P2-3	Forward	GAGAAGAAGAAGGTCAAATC	20	18508490–18508509
		Reverse	AGATAGAAACTGGAAAGGAG	20	18508680–18508698
	P2-4	Forward	AAGAAAGAGAGAGGGTATGTAG	22	18508712–18508733
		Reverse	TTGTTCTCTAATTGCTCTCC	20	18508902–18508921
BP3	P3-1	Forward	TCCATTCTGTCCTTATCTCTC	21	20166169–20166189
		Reverse	AATGAGTCAGAAATAGGATACG	22	20166347–20166368
	P3-2	Forward	GTCCCACTAACATCCTGCCTC	21	20170079–20170099
		Reverse	ACAAGCTGAAGTGCCACCTG	20	20170276–20170295
	P3-3	Forward	CTGACCATTAAACTCCCTGAAG	22	20170758–20170779
		Reverse	GCGATCTTATAGGAGGACCAG	21	20170941–20170961
	P3-4	Forward	CAGGAGAGGATAACACTTGG	20	20176073–20176092
		Reverse	TACTCCCTAGCTCACCTCTG	20	20176254–20176273
BP4	P4-1	Forward	TTTGCTCATCCTGCTTTC	18	22475946–22475963
		Reverse	TACTGGAGTGGCTTGTGG	18	22476064–22476081
	P4-2	Forward	TAGCACTTTCTGTTCTCTTG	20	22514747–22514766
		Reverse	CCAATACAAATAGTCAGCC	19	22514878–22514896
	P4-3	Forward	AATGTTTGAGCAGTGTAGAG	20	22527641–22527660
		Reverse	GAAACTCTCTGCCTCATG	18	22527777–22527794
	P4-4	Forward	GCTAAGAGTACTGGGAAGGGAC	22	22528860–22528881
		Reverse	GCTCTGAGTGGACCTTGCTAC	21	22529002–22529022
